# No Difference in Myosin Kinetics and Spatial Distribution of the Lever Arm in the Left and Right Ventricles of Human Hearts

**DOI:** 10.3389/fphys.2017.00732

**Published:** 2017-10-13

**Authors:** Divya Duggal, S. Requena, Janhavi Nagwekar, Sangram Raut, Ryan Rich, Hriday Das, Vipul Patel, Ignacy Gryczynski, Rafal Fudala, Zygmunt Gryczynski, Cheavar Blair, Kenneth S. Campbell, Julian Borejdo

**Affiliations:** ^1^Department of Cell Biology and Center for Commercialization of Fluorescence Technologies, University of North Texas, Health Science Center, Fort Worth, TX, United States; ^2^Department of Physics and Astronomy, Texas Christian University, Fort Worth, TX, United States; ^3^Department of Mathematics and Physics, Texas Wesleyan University, Fort Worth, TX, United States; ^4^Center for Neuroscience Discovery, Institute for Healthy Aging, University of North Texas, Health Science Center, Fort Worth, TX, United States; ^5^Center of Emphasis in Diabetes and Metabolism, Paul L. Foster School of Medicine, Texas Tech University Health Sciences Center El Paso, El Paso, TX, United States; ^6^Department of Physiology, University of Kentucky, Lexington, KY, United States

**Keywords:** cross-bridge orientation, heart ventricles, fluorescence polarization

## Abstract

The systemic circulation offers larger resistance to the blood flow than the pulmonary system. Consequently, the left ventricle (LV) must pump blood with more force than the right ventricle (RV). The question arises whether the stronger pumping action of the LV is due to a more efficient action of left ventricular myosin, or whether it is due to the morphological differences between ventricles. Such a question cannot be answered by studying the entire ventricles or myocytes because any observed differences would be wiped out by averaging the information obtained from trillions of myosin molecules present in a ventricle or myocyte. We therefore searched for the differences between single myosin molecules of the LV and RV of failing hearts *In-situ*. We show that the parameters that define the mechanical characteristics of working myosin (kinetic rates and the distribution of spatial orientation of myosin lever arm) were the same in both ventricles. These results suggest that there is no difference in the way myosin interacts with thin filaments in myocytes of failing hearts, and suggests that the difference in pumping efficiencies are caused by interactions between muscle proteins other than myosin or that they are purely morphological.

## Introduction

In terms of morphology and function whole ventricles are bilaterally different. Smaller force is necessary to pump blood into the pulmonary circulation by the right ventricle than into the systemic circulation by the left ventricle. It is conventionally thought that this difference arises from the dissimilarities of basic fiber structures of both ventricles or that differences arise already at the myocyte level (Belin et al., 2011). Transverse fibers in the free wall of the right ventricle (RV) are shared with oblique fibers in its inter-ventricular septum. The left ventricle (LV) is composed largely of oblique and circumferential fibers (Schwarz et al., 2013), which are known to be more mechanically efficient than the transverse fibers of RV (Sallin, 1969; Austin, 2007). The idea that morphological differences are responsible for functional differences has been reinforced by the fact that there have been no confirmed reports of differences of the heavy chain composition or conformations between β-myosin molecules in the LV and the RV. Further, myosin chains are expressed from the same genes (MYH6 for myosin β-isoform of ventricular Heavy Chain in humans, MYL6 for Myosin Essential Light Chain, MYL2 for Myosin Regulatory Light Chain, MYL3 for Myosin Alkali Light Chain) (Park et al., 2011; Huang et al., 2015).

However, the question of stronger pumping action of the LV vs. RV cannot be fully assigned to morphological differences until the alternative possibility, that the differences are due to a more efficient function of myosin or other myocyte proteins in the LV than the RV, is answered. The focus of this study is to test whether myosin function is he same or different in both ventricles of failing heart.

The question of stronger pumping action of the LV vs. RV can't be answered by investigating the whole ventricles because whole ventricles (or papillary muscles) contain a large number of myosins (10^11^–10^13^) (Bagshaw, 1982). Data obtained from such a large number gets averaged out, and all the kinetic information, i.e., the rate constants of the mechanochemical cycle of myosin molecule, is lost. In such experiments, the contribution of an individual myosin molecule has to be recognized and not averaged out (Elson, 2004, 2011). Similarly, if a large number of molecules are examined concurrently, the final distribution of myosin will be perfect Gaussian, irrespective of whether the data are taken from the left or right ventricle (Central Limit Theorem, Bracewell, 1965). Because of these technical difficulties, the question of whether individual actin and/or myosin molecules function in a different way in LV and RV has never been asked. Our ability to study a few molecules out of trillions present in a whole ventricle makes the question possible to answer. The object of our study, myosin, is particularly convenient target for investigation because its function can be characterized by the motion of its lever arm that is easily fluorescently labeled *In-situ* without any damage to its ATPase activity.

The data presented here was obtained from the ventricles from failing hearts. The reason for this is that failing ventricles are relatively easy to obtain (from patients undergoing heart transplant). While failing ventricles suffered variable amounts of damage, myocytes shortened after addition of contracting solution (Figure S2) and retained ATPase activity.

We measured the kinetics and distribution of few myosin molecules in the A-band of an isometrically contracting sarcomere. The A-band is a volume where a force-producing interaction between actin and myosin molecules takes place. Since molecular crowding plays an important role in *in-vivo* situations (especially in muscle where protein concentration is extremely high, Bagshaw, 1982), such measurements must be carried out under conditions as closely as possible resembling the situation *in-vivo*, in other words *In-situ* in the working ventricle (Minton, 2001; Mourao et al., 2014). We show that the kinetics and the steady-state distribution of myosin were the same in contracting left and right ventricles from the failing (HF) human heart. The results suggest that there is no difference in the way myosin interacts with thin filaments in ventricles of failing hearts, and that the difference in ventricular function is caused by proteins other than myosin or by morphological differences between ventricles.

### Principle of measurements

A myosin head consists of a globular head and α-helical lever arm. The lever arm is believed to undergo a large “swing” resulting from actin-activated hydrolysis of ATP by the myosin head. The swing is responsible for a relative sliding of myosin and actin containing filaments and force development during contraction. The essential Light Chain (LC) of myosin resides on the lever arm and therefore is a convenient site to monitor the swing (orientation of the lever arm). Steady State Fluorescence Anisotropy (SSFA) is a well-known method to monitor the orientation. Therefore, the values of SSFA in different physiological states of muscle are an indication of orientation changes during XB cycle.

**Figure 1:**
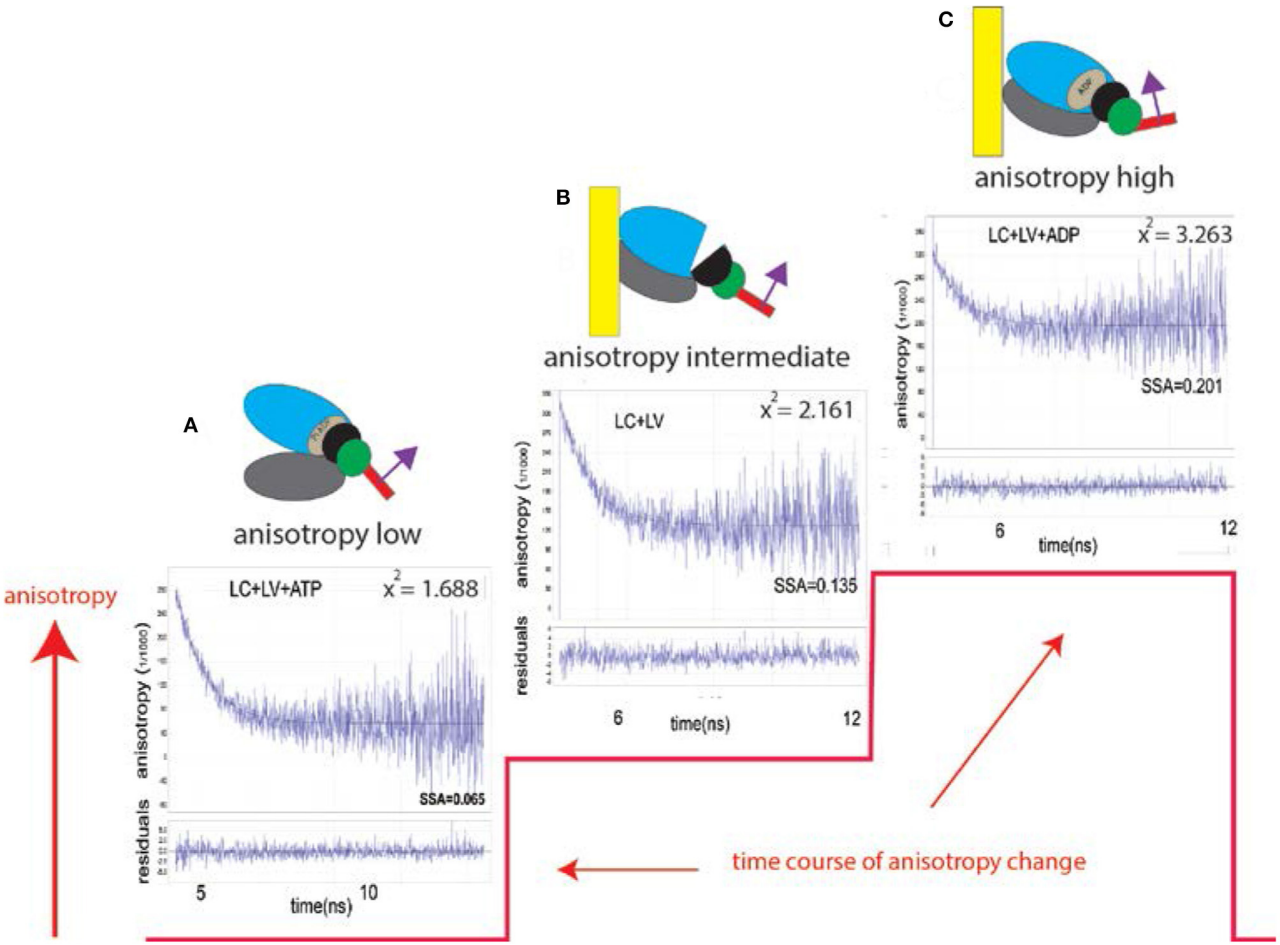
Changes of conformation of a cross-bridge during the contractile cycle are reflected by changes of anisotropy of the myosin lever arm (red line). The kinetics of orientation changes of a lever arm is a characteristic property of muscle. The instrument used to measure anisotropy is shown in Figure S1. Fluorescent LC sits on a lever arm of a cross-bridge (red rectangle). LC orientation is indicated by the magenta arrow; **(A)** XB is originally free of actin. Lower and upper 50 KDa domains of myosin XB (gray and blue, respectively) are separated. The lever arm is facing down. Steady State Fluorescence Anisotropy (SSFA) of the lever arm is low. **(B)** The anisotropy of LC+LV (rigor) complex (apo form of ventricular XB is bound to actin filament). Lower and upper 50 KDa domains of XB are closed. The anisotropy assumes intermediate value (SSFA = 0.135); **(C)** Interaction between XB containing ADP and actin. Lower and upper 50 KDa domains of XB are closed the anisotropy of a complex assumes the highest value (SSFA = 0.201). This is consistent with recent report (Wulf et al., 2016). The lower plot in each anisotropy graph is a residual. X^2^ indicates goodness of non-linear fit to a thin black line. 0.1 mg/ml myocytes from the left ventricle, 2 mM MgADP, 5 mM MgATP. Excitation at 630 nm by Fianium supercontinuum white light source. The emission was observed at 665 nm through long pass 650 nm filter by a fast microchannel photomultiplier tube.

To monitor the orientation of LC during ventricle contraction, LC was expressed in *E. coli*, labeled with fluorescent dye and exchanged with native ventricular LC. Figure 1 illustrates how the anisotropy (measure of the orientation) of labeled LC reflects the kinetics of a myosin cross-bridge (XB).

**Figure 2:**
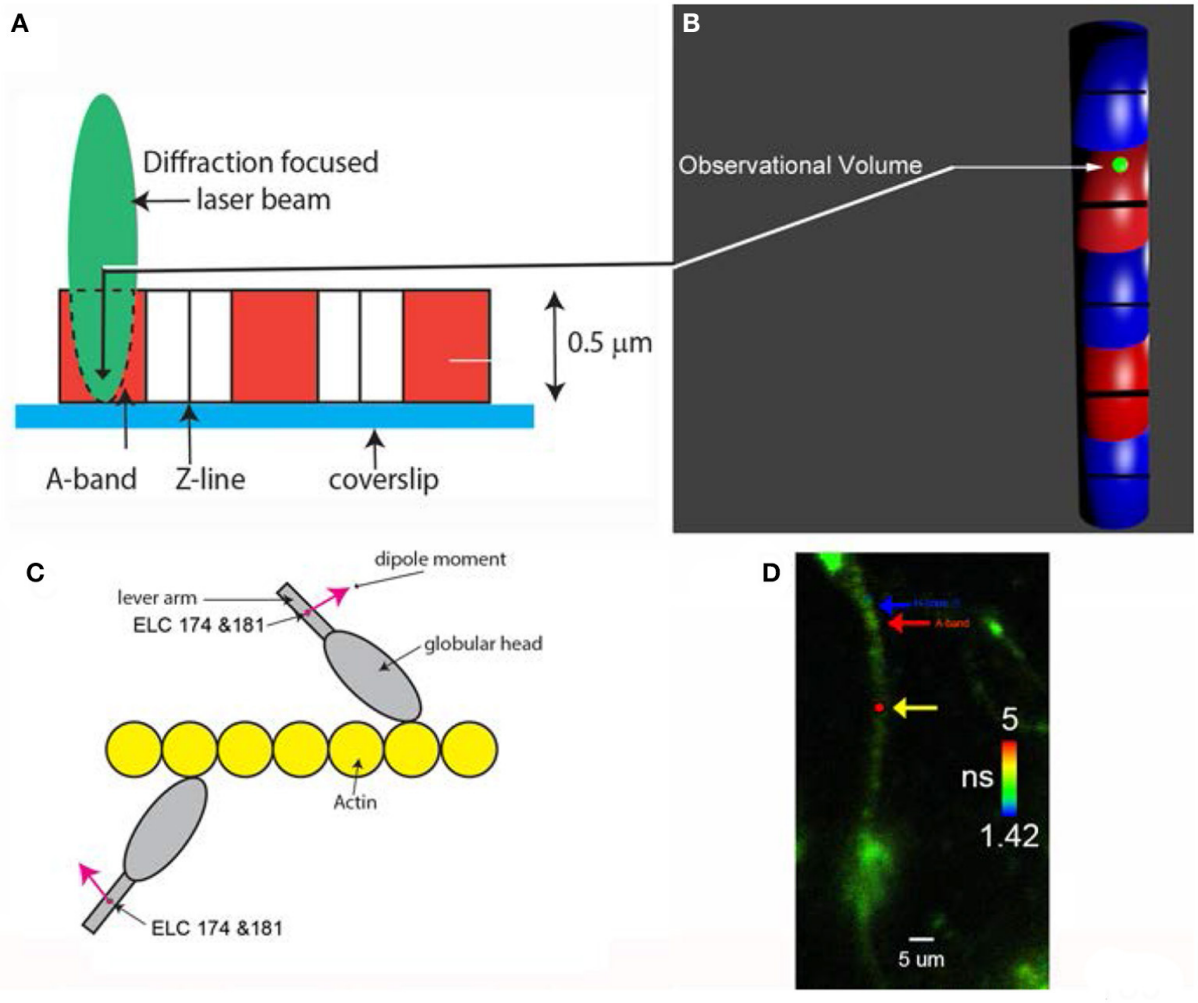
**(A)** A diffraction limited laser beam (green ellipsoid) is focused on an isolated myocyte sitting on a coverslip. A confocal microscope sees only the Observational Volume (OV, outlined by a broken line). The A-band (red) is the only region where the interaction between actin and myosin occurs. **(B)** Fluorescence is collected from the OV shown here in 3D as a green sphere imbedded in the A-band. It is projection of the confocal aperture on the image plane. The diameter of the OV (0.5 μm) is equal to the diameter of the confocal pinhole (50 μm) divided by the magnification of the objective (100x). Myosin within the A-band is fluorescently labeled (red). The I-bands are non-fluorescent (blue). Thick black lines are the H-zones; thin black lines are the Z-bands. The myocyte contracts (i.e., develops normal force), but does not shorten because it is cross-linked (see Methods); **(C)** One in approximately one thousand myosin molecules within the thick filament is labeled at LC Cys 174 and Cys 181. The transition dipole moments the dye of two labeled myosins are marked in magenta. Emission dipoles of the dye attached to these two cysteines point in the same direction. Labeling of only 1 in a 1,000 myosins ensures that there are only 6 myosin molecules in the OV (see Methods). Actin monomers (yellow) are non-fluorescent. **(D)** Fluorescent lifetime image of a myocyte from a non-failing human RV in rigor myocytes were imaged by fluorescent lifetime imaging because a lifetime image is of better quality than a confocal image. Nevertheless, the quality of the image is poor because of intentionally inefficient labeling (see Methods). On the average only ~6 myosin molecules/half-sarcomere were labeled with the fluorophore (see Methods). The color bar indicates lifetime (in nanoseconds) of a given pixel. The non-fluorescent part (H-zone) is pointed to by the blue arrow. Data was collected from the red spot (pointed to by the yellow arrow). The data was collected only from the part of a myocyte which was aligned vertically (thus it was not collected from the A-band pointed to by the red arrow). The scale bar is 5 μm. The red dot (pointed by the yellow arrow) is the 2D projection of the OV. The image has not been contrast enhanced.

To observe a small number of myosin molecules *In-situ* it is necessary to carry out experiments on the A-band of the isolated myocytes from human ventricles. The A-band is the volume where actin and myosin interact to produce contractile force. Myocyte A-band contains ~10^4^ myosin molecules, still too many to obtain kinetic information. To reduce this number, myosin was intentionally labeled inefficiently with fluorescent probe (i.e., only 1 in a 1,000 myosins were labeled). Myocytes were cross-linked with zero-length cross-linker to eliminate any movement during contraction while preserving ATPase activity. Figure 2 explains how the experiments were carried out.

## Materials and methods

### Chemicals and solutions

All chemicals were from Sigma-Aldrich (St Louis, MO). The fluorescent dye SeTau-647-mono-maleimide was from SETA BioMedicals (Urbana, IL). The glycerinating solution contained: 50% glycerol, 150 mM KCl, 10 mM Tris-HCl pH 7.5, 5 mM MgCl_2_, 5 mM EGTA, 5 mM ATP, 1 mM DTT, 2 mM PMSF, and 0.1% β-mercaptoethanol. The rigor solution: 50 mM KCl, 10 mM Tris-HCl pH 7.5, 2 mM MgCl_2_. The EDTA-rigor solution: 50 mM KCl, 10 mM Tris-HCl pH 7.5, 5 mM EDTA. The Ca-rigor solution: 50 mM KCl, 10 mM Tris-HCl pH 7.5, 2 mM MgCl_2_, 0.1 mM CaCl_2_. The contracting solution: 50 mM KCl, 10 mM Tris-HCl pH 7.5, 5 mM MgCl_2_, 0.1 mM CaCl_2_, 5 mM ATP, 20 mM creatine phosphate and 10 units/ml of 1 mg/ml creatine kinase. The relaxing solution: 50 mM KCl, 10 mM Tris-HCl pH 7.5, 5 mM MgCl_2_, 5 mM ATP, 2 mM EGTA.

### Preparation of human essential light chain

LC was genetically modified to contain two thiol groups (rather than 1). This makes it possible to attach two molecules of the dye to the light chain and double the extinction coefficient of the probe. To modify ELC, the human fast skeletal muscle ELC was subcloned into pQE60 vector to produce pQE-ELC expression vector. The LC cDNA insert in pQE60-LC construct contains one *Cys* at amino acid position 181. We have introduced *Cys* in place of *Gly* at amino acid position 174 by site directed mutagenesis to generate pQE60-LCG174C expression vector. The resulting pQE60-LCG174C expression vector contained two *Cys* residues at amino acid positions 174 and 181. The pQE60-LCG174C construct was generated by PCR-based site directed mutagenesis using the QuickChange kit from Strata Gene (La Jolla, CA), pQE60-LC template plasmid, and two complimentary primers:

F-hLCG174C: 5′-gaagtggaagccctgatggcatgtcaagaagactccaatggctgc-3′ and

R-hLCG174C: 5′-gcagccattggagtcttcttgacatgccatcagggcttccacttcttcc-3′. The sequence of the plasmid pQE60-LCG174C was confirmed by DNA-sequencing of both strands of the entire plasmid. Afterwards, the plasmid pQE60-LCG174C was introduced into *Escherichia coli* M15 cells (Qiagen). The expressed recombinant proteins were purified on Ni-NTA-Agarose column and confirmed by immunoblotting with human LC monoclonal antibody. (We also tried to cross-link two thiols with bifunctional rhodamine in order to immobilize the dye, a procedure originally developed by Irving's group (Corrie et al., 1998, 1999; Hopkins et al., 2002; Lewis et al., 2012), but in our hands this strategy was unsuccessful).

### Preparation of ventricles

Samples of human myocardium were collected at the University of Kentucky using procedures that were approved by the local Institutional Review Board. The human cardiac samples used in this study were acquired using the collection protocol described by Blair et al. (2016) hearts procured from patients undergoing cardiac transplants at the University of Kentucky and from organ donors who did not have heart failure. Some samples were cut into strips of ventricular walls and analyzed for tension and ATPase by Dr Campbell. Whole ventricles were then given to a researcher immediately after being removed from the patient. Ventricular tissue was dissected into ~500 mg samples and snap-frozen in liquid nitrogen within 30 min of being removed from the patient. These samples were then stored in the vapor phase of liquid nitrogen at −150°C until use. The University of Kentucky Institutional Review Board approved all procedures, and subjects gave informed consent (IRB 08-0338-F2L).

All samples were passed to a researcher as soon as they were removed from the patient and snap-frozen in liquid nitrogen within a few minutes. Samples were shipped to UNTHSC on dry ice. Immediately upon arrival in Fort Worth, they were tied to wooden sticks (in a cold room) and placed for 24 h in glycerinating solution at 0°C. After 24 h, the glycerinating solution was replaced with a fresh solution and placed at −20°C. Myocytes were made from glycerinated hearts after a minimum of 2 weeks at −20°C.

### Preparation of myocytes from a ventricle

Human ventricular myocytes were used in all experiments. Use of myocytes eliminates problems associated with the non-uniformity of labeling of sarcomeres and assures better access of ligands to the contractile proteins. Myocytes were prepared from ventricles as follows: ATP present in the glycerinating solution was first removed without causing contraction by washing ventricles three times for 30 min with ice-cold EDTA-rigor solution (50 mM KCl, 10 mM Tris-HCl pH 7.5, 5 mM EDTA) followed by extensive washing with the Ca-rigor solution (50 mM KCl, 10 mM Tris-HCl pH 7.5, 2 mM MgCl_2_, 0.1 mM CaCl_2_).They were homogenized in the Ca-rigor solution (to avoid forming foam upon turbulent stirring when homogenized in EDTA-solution.) in the Cole-Palmer LabGen 125 homogenizer for 10 s followed by further 10 s homogenization after a cool down period of 30 s.

### ATPase assay

The measurements were carried out using Anaspec (Fremont, Ca) Sensolyte MG Phosphate Colorimetric Assay at 30°C in a 96-well plate and read on a microplate reader at 650 nm, with and without cross-linker EDC. The myocytes (1 mg/ml) were incubated in EDC as described above. The reaction mixture of 50 mM KCl, 5 mM MgCl_2_, 20 mM EDC, 5 mM ATP in Ca-rigor along with 1 mg/ml myocytes was made to 80 μl in volume to which 20 μl Malachite Green (MG) reagent was added. Samples were ran in triplicate. Readings were taken for 30 min at 2 min. intervals. A graph was plotted to calculate the Pi released per s.

### Cross-linking

One milligram per milliliter myocytes were incubated for 20 min at room temperature with 20 mM water soluble cross-linker 1-ethyl-3-[3-(dimethylamino)-propyl]-carbodiimide (EDC) in the Ca-rigor solution. It is known that cross-linking of skeletal myocytes does not affect tension or ATPase (Herrmann et al., 1994; Bershitsky et al., 1997; Barman et al., 1998; Tsaturyan et al., 1999). The reaction was stopped by adding 20 mM DTT. To check whether cross-linking does not affect ATPase of cardiac MFs, ATPase was measured independently by two researchers. ATPase varied between 0.028 ± 0.001 and 0.032 ± 0.007 mols Pi/s/mol myosin, in agreement with (Alpert and Gordon, 1962). 1 In every case *R*^2^ was 0.98 or 0.99. The ATPase was slightly higher for the RV than the LV. The pH of the solution (7.5) remained unchanged throughout the 20 min reaction.

### LC labeling

Recombinant LC (G174C-hLC) was labeled at positions 174 and 181 with three-fold excess of SeTau 647 Maleimide. SeTau647-maleimide was chosen to label SH groups on LC because it is excited in the red and hence bypasses most contributions of autofluorescence (Lakowicz, 2006). SeTau is also very resistant to photobleaching (the initial rate of photobleaching was 2.4 s^−1^) because of nano-encapsulation of the squaraine moiety of the dye chromophore system in a mixed aliphatic-aromatic macrocycle. SeTau has a large Stokes shift (44 nm), very high extinction coefficient (200,000 M^−1^ cm^−1^) and quantum yield (0.65). Its overall fluorescence intensity was 4.2 times larger than the fluorescence intensity of Alexa647. The reaction was allowed to occur overnight. Excess dye was removed by centrifugal filtration followed by overnight dialysis. Using two SeTau molecules per molecule of LC increases overall extinction coefficient to an extraordinary 400,000 M^−1^ cm^−1^. It is possible that SeTau resides on two different myosin molecules rather than single myosin, but this is of no consequence because ultimately there are ~6 molecules of SeTau in the OV (see below). The dyes were well-immobilized by myocytes in rigor and in the presence of MgADP (see below), and their combined transition dipoles have a well-defined orientation.

### Exchange of labeled LC into myocyte myosin

Following labeling of recombinant peptide with SeTau-647 Maleimide, 5 nM of this protein was exchanged with the native LC of ventricular myosin in the exchange solution containing 15 mM KCl, 5 mM EDTA, 5 mM DTT, 10 mM KH_2_PO_4_, 5 mM ATP, 1 mM TFP, and 10 mM imidazole, pH 7 (Duggal et al., 2015; Huang et al., 2015). The reaction was allowed to occur at 30°C for 10 min. This ensured that the exchange was inefficient (i.e., that only a small fraction of myosin carried fluorescent label). Two milligram per milliliters of freshly prepared myocytes were used.

### Data collection

The fluorescence was measured by a PicoQuant MT 200 time-resolved fluorescence instrument coupled to an Olympus IX 71 microscope. Before each experiment, the instrument was calibrated by measuring fluorescence of an isotropic solution of a dye with a long fluorescence lifetime (rhodamine 700, excitation at 635 nm). Such fluorescence is completely polarized (steady-state anisotropy *r* = 0.400, I. Gryczynski, unpublished) because the dye with a long lifetime has no time to reorient itself during a ns short laser pulse. This procedure makes sure that 100% of emitted light was detected by a parallel (∥) channel. If it was not, the prism position was adjusted until it was. Rotating the birefringent prism by 90° made sure that the 100% of emitted light was detected by a perpendicular (⊥) channel. The beam was focused by an Olympus x60, NA = 1.2 water immersion objective on the A-band of a myocyte. The fluorescence intensity was collected into 10 μs bins for 20 s (i.e., there were 2,000,000 bins). To smooth the data, 1,000 bins were averaged into 2,000 bins yielding overall time resolution of 10 ms. Polarization of fluorescence (PF) and steady-state anisotropy (r) were computed from the orthogonal components of fluorescent light. The autocorrelation function (ACF) and the plot of PF vs. frequency with which it occurs over 20 s (histogram), was constructed from these 2,000 measurements. Approximately 20–30 myocytes were examined in each ventricle.

### Estimating the number of observed myosin molecules

To determine the number of observed myosin molecules, it was necessary to measure fluorescence intensity associated with one molecule of SeTau. This number was determined by Fluorescence Correlation Spectroscopy (FCS). The autocorrelation function at delay time 0 of fluctuations of freely diffusing SeTau molecules entering and leaving the OV is equal to the inverse of the number of molecules contributing to the fluctuations N = 1/ACF(0) (Magde et al., 1974; Elson, 1985, 2007). Fifteen nanomolars SeTau was illuminated with 1–70 μW of laser power at 635 nm. A calibration curve was constructed by plotting the power of the laser vs. the rate of photon arrival per molecule of the dye. From this curve it was determined that the number of photons/s collected from a single SeTau molecule was ~900.

**Figure 3:**
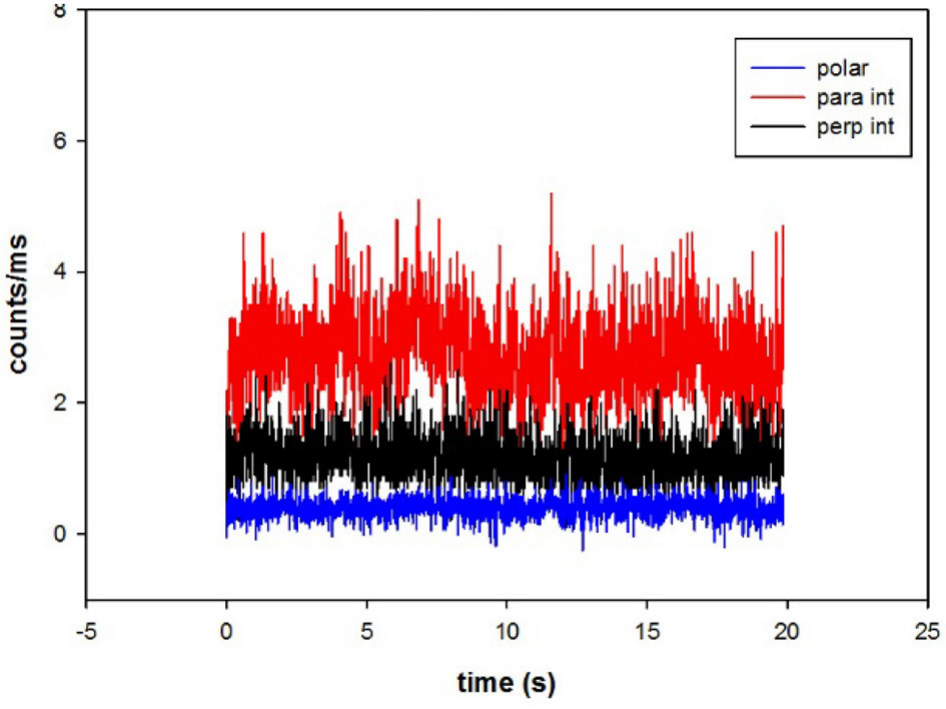
The time course of intensities of fluorescent light from myosin. Fluorescent intensities are polarized parallel (red) and perpendicular (black) with respect to the axis of the myocyte. Polarization of fluorescence is shown in blue. The average polarization of fluorescence was 0.400 ± 0.158. The signal from the OV of a left ventricle in the presence of MgADP.

A typical fluorescent signal obtained from ventricular myocyte is shown in Figure 3.

The average number of observed photons per second in a parallel channel was 2,731 ± 645 (Figure 3, red). The average number of photons per second in perpendicular channel (black) was 1,161 ± 364. The average total fluorescence intensity was I_∥_ + 2^*^I_⊥_ = 5,053 ± 1,373 counts/s. Therefore, the average number of observed myosin molecules was 5,053/900 = 5.6. However, it should be noted that as long as the number of molecules is mesoscopic, the exact number does not matter. The same results would have been obtained observing 6 or 60 molecules.

### Time resolved anisotropy measurements

FT300 fluorescence lifetime spectrometer (PicoQuant GmbH, Germany) was used for time-resolved measurements. The muscle was excited by Fianium supercontinuum white light (Fianium Ltd, Whitelase SC400-4). The white light was passed through a monochromator set at 635 nm at 10 MHz repetition rate. The emission was passed through long pass 650 nm filter and detected by a fast microchannel photomultiplier tube. The resolution was kept at 4 ps per channel and the pulse width was less than 100 ps. Fluorescence intensity decays were collected while orienting the emission polarizer in vertical and horizontal position respective to the vertically oriented excitation polarizer for measuring anisotropy. The vertical (parallel) and horizontal (perpendicular) intensity decays were used to calculate the time dependent anisotropy using the equation (*t*) = (*I*_*Parallel* (*t*)−*I*_*Perpandicular* (*t*))/(*I*_*Parallel* (*t*)+2*I*_*Perpandicular* (*t*)).

The obtained anisotropy decay was analyzed using Fluofit 4.0 program provided by PicoQuant and was fitted using formula *r*(*t*) = ∑ *R*_*ie*^∧^(−*t*/Ø_*i*) where r is the total anisotropy, R_i_ is the fractional anisotropy amplitude associated with ith component, Ø_*i* is the i-th rotation correlation time and t is the time in nanoseconds. SSFA is the mean of r at all times until the time after which r remains constant (12 ns).

### Statistical analysis

Comparisons between groups were performed using the unpaired Student's *t*-test by Origin v.8.6 (Northampton, MA). Origin was also used to fit the data by a non-linear routine, calculate histograms, and autocorrelation functions. The significance was defined as *P* < 0.05.

### Problems

The chief difficulty in obtaining single molecule data from a ventricle *In-situ* lies in assuring adequate Signal/Noise ratio. Adequate S/N is possible when observing a single myosin molecule *in-vitro* (Forkey et al., 2003, 2005). However, *in-vitro* data cannot be viewed as originating from independent motors (Walcott et al., 2012) (Pate and Cooke, 1991; Baker et al., 2002). *In-situ* data from a ventricle contains significant contributions from the background. In addition to the very strong autofluorescence due to an extremely dense environment (Bagshaw, 1982), the background consists of a constant fluorescence coming from myosin that is always present in the OV. In contrast to the regular FCS, the myosin molecules do not translate and fluorescent signal does not fluctuate between zero and maximum. In our experiments only the PF fluctuates around the mean. Moreover, to avoid photobleaching the laser beam cannot be focused on the same spot for much more than 20 s.

## Results

### Kinetics of contraction of failing ventricles

We emphasize again that the data presented here was obtained from the ventricles from HF hearts. When cross-linked, they do not shorten, but retain full ATPase activity 0.040 ± 0.004 s^−1^/mol; 0.034 ± 0.004 s^−1^/mol for control and cross-linked respectively for the LV, and 0.033 ± 0.003 s^−1^/mol; 0.037 ± 0.004 s^−1^/mol for control and cross-linked respectively for the RV.

**Figure 4:**
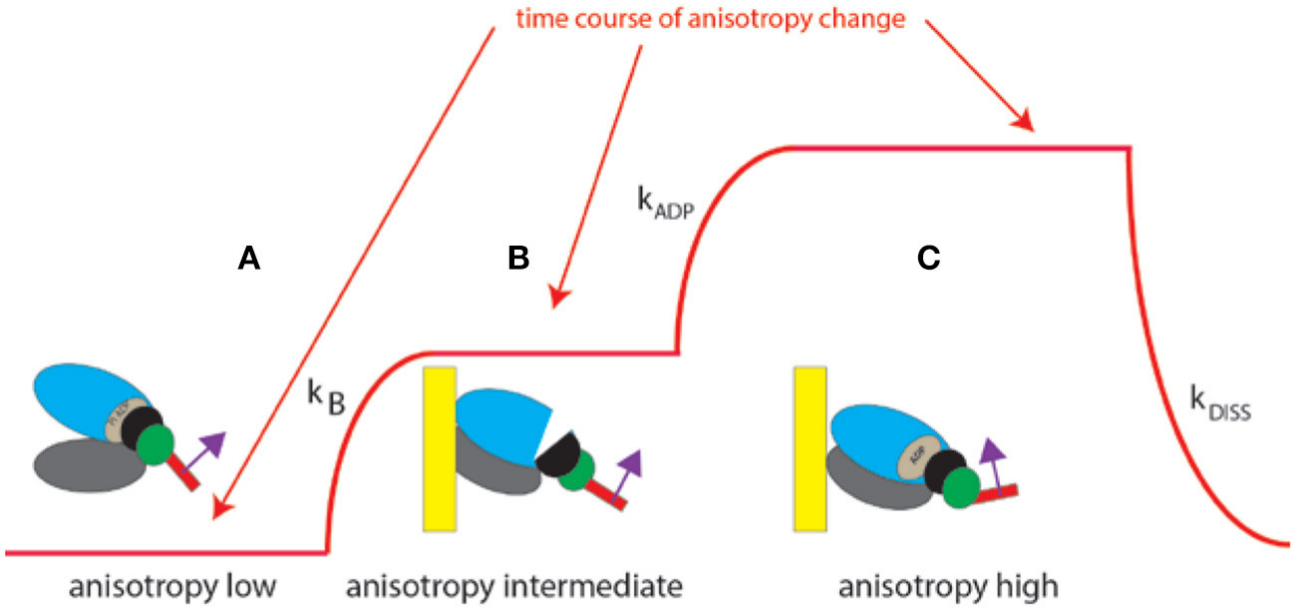
Conformational transitions of 6 XBs during contraction of a ventricle. Red line indicates change of anisotropy. **(A)** Binding of cross-bridges to a thin filament of a ventricle is characterized by the rate constant k_B_; **(B)** transition from apoenzyme to holoenzyme form of myosin is characterized by the rate constant k_ADP_; **(C)** dissociation of a cross-bridge from a thin filament characterized by the rate constant k_DISS_.

The experiment begins by placing isolated myocyte on an ethanol cleaned cover slip. Because we observe ~6 myosin molecules (and not a single one), the changes of anisotropy occur gradually (Figure 4, and not in steps as illustrated in Figure 1 which shows changes for a single molecule). The time course of SSFA can be simplified to a scheme shown as a Figure 4, red line. The cycle begins when XB is dissociated from a thin filament where SSFA is low (A). Binding to a thin filament causes a small increase in SSFA (B) transition (A → B). Dissociation of Pi and assumption of actin-myosin-ADP complex (transition from apoenzyme to holoenzyme (B → C) causes further increase in SSFA (C) in accordance with (Coureux et al., 2004). Finally, dissociation of myosin from actin (C → A) occurs with the rate k_DISS_.

To extract the rate constants from fluorescence data we used the method first introduced by Magde and Elson (Magde et al., 1974; Elson, 1985, 2007). In this method Autocorrelation Function (ACF) of fluctuations of anisotropy is calculated. ACF is an average of the sum of products of the instantaneous values of anisotropy and the values of anisotropy delayed by delay time τ ranging from 0 to 1 s. The process of calculating ACF is shown in Figure S3. The analytical form of ACF of the three-state process is very complex and is fully described in Mettikolla et al. (2011).

**Figure 5:**
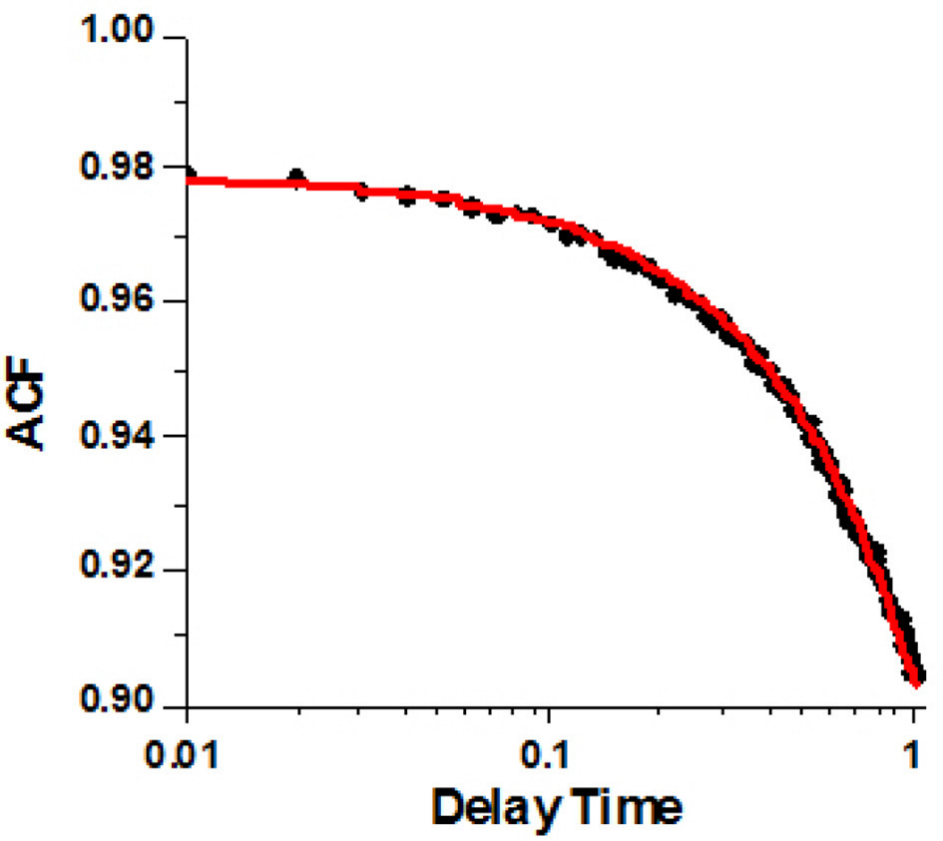
A typical autocorrelation function of the lever arm fluctuations of contracting cardiac myosin in HF RV. Filled circles are the data. Red line is non-linear fit to analytical solution described in Mettikolla et al. (2011).

A typical example of the ACF data from a HF ventricle is shown in Figure 5. The non-linear fit to the analytical form of ACF is shown as the red line. The experimental data clearly indicates a two-state process. XB binding to actin (rate k_B_) is too fast to be reflected in the experimental points. This is due to the limited time resolution of the instrument. It collects photons every 10 μs, but in order to decrease the noise, the 2 M data points are packed in 2,000 bins, 1,000 points per bin, Figure S3. Thus, the instrumental response time is 10 ms. We cannot detect processes faster than 100 Hz (s^−1^).

We analyzed data from 6 HF ventricles. One ventricle was from a patient suffering from non-compaction. Data from this ventricle was eliminated. Table 1 shows averages from five remaining ventricles. There was no statistical difference between LV and RV for either rate constant. The data from individual ventricles is shown in Figure S4.

**Table 1:** Differences between the kinetic rate constants of LV and RV of heart failure ventricles.

**HF Contraction**	**k_ADP_**	**k_DISS_**
HF LV	1.69 ± 0.05	2.24 ± 0.06
HF RV	1.72 ± 0.04	2.36 ± 0.20

*The results are averages of 27 experiments on LVs and 26 experiments on RVs. Errors are SD. Overall, the differences in the values of HF LV and RV were not significant*.

### Spatial distribution of XB from failing ventricles during contraction

**Figure 6:**
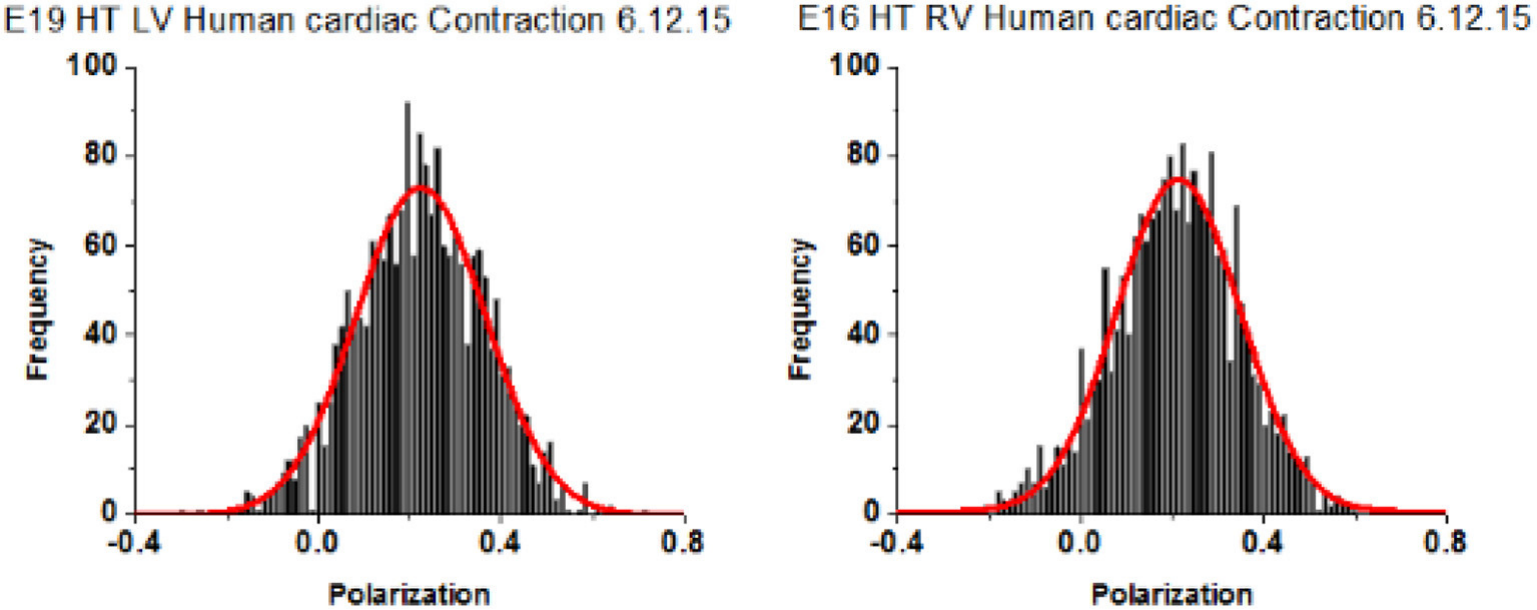
Spatial distribution of lever arm angles of cross-bridges of contracting HF ventricles. The orientation of ~6 cross-bridges in 27 different preparations of LV and 26 preparations of RV Data shows no difference in FWHM. Bars are data. Red line is the best fit.

A characteristic feature of a distribution of orientations is Full Width at Half Maximum (FWHM) [it is related to Standard Deviation, *SD*; for Gaussian distributions FWHM = 2Sqrt(2ln2) = 2.355 *SD*]. The distribution of polarizations was Gaussian. The differences between FWHM's of polarization of fluorescence of different populations of ventricles are statistically interpretable only when the signal from each half-sarcomere is similar. This is because the relative value of FWHM of a strong random signal is small relative to a weak signal (Midde et al., 2013). To comply with this requirement, the power of a laser was adjusted within 1–2 μW of the mean. Examples of measurements of 27 experiments from LVs and 26 experiments from RVs are shown in Figure 6. The distributions are displayed here as histograms (plots of frequency of occurrence of a given orientation.

Polarization of Fluorescence (PF) rather than steady-state anisotropy is used here to describe orientation of the lever arm because it has been used routinely to measure conformation of XBs (Dos Remedios et al., 1972; Nihei et al., 1974; Tregear and Mendelson, 1975; Morales, 1984; Hopkins et al., 1998, 2002; Sabido-David et al., 1998). PF is closely related to the SSFA by SSFA = 2PF/(3-PF). PF is the difference between ∥ and ⊥ components of the fluorescent light emitted by the dye, normalized by their sum.

All data is summarized in Table 2. The distributions show no differences in the value of FWHM. AR^2^ values indicate how well the fitted curve (red) matches a perfect Gaussian (a perfect fit has an AR^2^ value of 1).

**Table 2:** The FWHM of distribution of lever arm angles of cross-bridges in the A-band of contracting LVs and RVs myocytes from HF human heart.

**Contraction**	**FWHM**	**AR^2^**
HF LV	0.390 ± 0.03	0.853 ± 0.096
HF RV	0.381 ± 0.01	0.889 ± 0.078

*The results are averages of 27 experiments on LVs and 26 experiments on RVs. Errors are SD. By conventional criteria, the statistical significance of difference of FWHM was not statistically significant (t = 0.691, P = 0.505) within 10 degrees of freedom. AR^2^measures goodness of fit (closer to 1 the better).The data obtained from individual ventricles is shown in the Figure S5*.

## Discussion

We obtained data from a few myosin molecules *In-situ* in working myocytes. This avoids complications due to the fact that the LV and RV show differences in the basic fiber structures. Further, data was collected under *in situ* conditions thus taking into account molecular crowding and packing of myosin in thick filaments, which play an important role in crowded systems such as muscle. Taken together, our results suggest that there is no difference in the way myosin interacts with thin filaments in ventricles from HF hearts, and suggests that the difference in pumping efficiencies of ventricles is morphological or that other muscle proteins impose differences: earlier work showed interventricular differences in myocyte function in experimental congestive heart failure of rats. Failing RV and LV myocytes displayed similar decrease in development of maximal force but failing LV myocytes were less Ca^2+^-sensitive (than failing RV myocytes). There were also differences in expression and activation of PKC-α and in phosphorylation of cTnI and cTnT (Belin et al., 2011). A possible reason for the difference between this work and (Belin et al., 2011) is that different species were examined. Another possibility is that individual molecules were examined here, whereas Belin et al. looked at whole myocytes.

However, the question still remains whether in non-failing (NF) ventricles the difference between LV and RV is also present. In NF ventricles, unlike in HF ventricles, many differences between whole tissues were observed. Differences have been reported in the ATPase activity (Krug et al., 1987), in the α-heavy chain composition and velocity of contraction (Brooks et al., 1987), energy usage (Itoya et al., 1996; Carlsson et al., 2012), amounts of proteins (Cadete et al., 2012), synthesis and degradation of myosin chains (Samarel, 1989), in the contractile performance in dilated cardiomyopathy (McMahon et al., 1996), and congestive heart failure (Belin et al., 2011), in ventricular development (Rosenquist, 1970; de la Cruz et al., 1977; Tam et al., 1997; Mjaatvedt et al., 2001; Buckingham et al., 2005), in the expression of transcription factors expressed during the development (Srivastava et al., 1997) and in the mRNA and miRNA gene expressions (Drake et al., 2011). Our earlier results revealed the functional differences between healthy ventricles in the hearts of mice. (Nagwekar et al., 2014) and rabbits (Nagwekar et al., 2016). This question cannot be answered as yet. We don't have enough molecular data from NF ventricles to make a statistically valid conclusion (Figure S6). However, it must be emphasized that the fact that the existing NF data suggests functional differences between healthy ventricles does not imply that lack of differences is somehow normalized by heart failure.

It must be mentioned that the orientation of the lever arm was measured using recombinant light chain exchanged with endogenous LC. This leaves open the possibility that the data may not reflect exactly on the interactions of endogenous LC which carries no fluorophores. Post-translational modifications are unlikely to affect the results because in the mutated clone G has been shown to have Cys at amino acid position 147 by DNA sequencing. Therefore, expression of this mutated plasmid is expected to produce G147C isoform of the protein. The most obvious post-translational modification, formation of Cys-Cys link between Cys 147 and 181, is impossible because sample was pre-washed with DTT (ll. 265). The isoform shift will occur upon substituting Gly for Cys and the overall negative charge will decrease.

When we obtain statistically valid results for animal NF ventricles, it will have important clinical implications. We will then be able to make comparisons between LVs and RVs from HF and NF ventricles. If it turned out that LV from NF hearts are different from LVs from HF hearts or that RVs from NF hearts are different from RVs from HF hearts it would open the possibility of manipulation of rate constants and distributions to make the ventricular function of HF and NF ventricles similar. We think that the precise modifiers of rate constants and distributions will be found to minimize the differences between NF and HF ventricles. Myosin is a particularly convenient target for investigation because it is an allosteric molecule, where the actin binding domain, the nucleotide binding pocket, and the converter domain are in constant communication (Spudich, 2014). It should therefore be possible to find allosteric effectors that modify its function. The binding of a small molecule to myosin may perturb its function, specifically the efficiency of its interaction with actin and thus power output of a ventricle (Spudich, 2014). Any drug that potentiates of power output is likely to have a positive effect on a ventricle pumping too weakly. Likewise, specific inhibitors that affect force development (by altering the rate constants) to reduce power and velocity of muscle shortening are likely to be effective.

## Conclusions

- We looked for the differences between myosin cross-bridges of the LV and RV.
- Experiments were done *In-situ* thus accounting for molecular crowding.
- Cross-bridges from the left and right ventricles of contracting HF hearts were kinetically identical.
- Cross-bridges from the left and right ventricles of contracting HF hearts were identically distributed in space.
- The difference in pumping efficiencies between LV and RV of HF hearts are caused by muscle proteins other than myosin or are morphological.

## Author contributions

JB formulated the problem, designed experiments, and helped writing the paper. DD and JN carried out experiments. SRa and SRe RF, VP, and RR carried out computations. HD did genetic manipulation of myosin. IG and ZG did spectral analysis. CB and KC did initial test of the ventricles in Lexington and supplied them to JB.

### Conflict of interest statement

The authors declare that the research was conducted in the absence of any commercial or financial relationships that could be construed as a potential conflict of interest.

## Abbreviations

ACF: AutoCorrelation Function
EDC: ethyl-3-[3-(dimethylamino)-propyl]-carbodiimide
LC: human Essential Light Chain
FCS: Fluorescence Correlation Spectroscopy
HF: Heart Failure Ventricles. Ventricles from the hearts of patients before heart transplant surgery
LV: Left Ventricle
NF: Ventricles from deceased donors who had Non-failing Heart
OV: Observational Volume
PF: Polarization of Fluorescence
RV: Right Ventricle
*SD*: Standard Deviation
SSFA: Steady State Fluorescence Anisotropy
XB: myosin Cross-Bridge.
