# Supplementary material for: No Difference in Myosin Kinetics and Spatial Distribution of the Lever Arm in the Left and Right Ventricles of Human Hearts
Source: Front Physiol. 2017 Oct 13;8:732. doi: 10.3389/fphys.2017.00732 (PMC5645524; doi:10.3389/fphys.2017.00732)
Supplement: Supplementary file 1 [file DataSheet1.PDF]

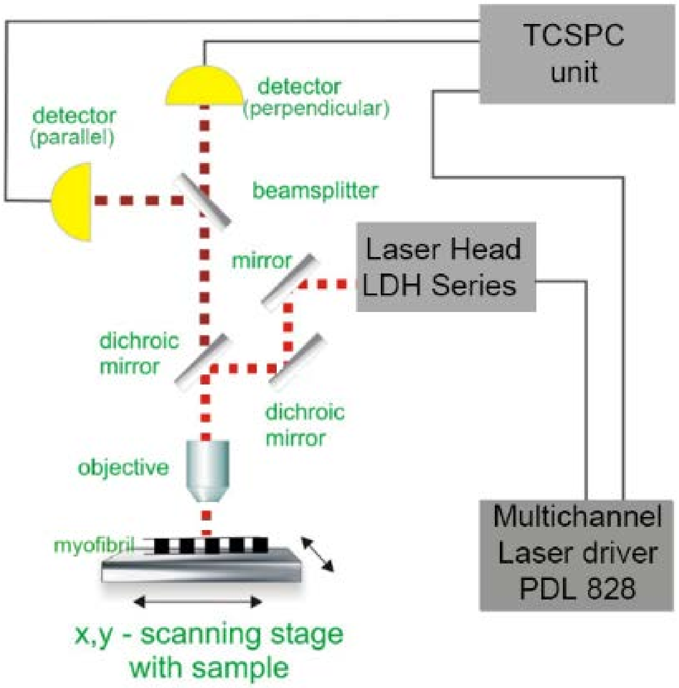

Fig. S1. Instrument *(PicoQuant MT200). Exiting pulsed (red light) is directed by an objective to an A-band of a sarcomere of an isolated myofibril. X-Y scanning is disabled. Exciting light is provided by a pulsed LDH series laser head. Fluorescent light (dark red) is collected by the same objective, filtered by dichroic mirror and an additional long-pass filter (not shown) and projected onto a polarizing beam-splitter which splits the light into parallel and perpendicular components. Filtered light is collected and photons are counted by the Time Correlated Single Photon Counter unit, binned and transferred into separate computer for processing.*

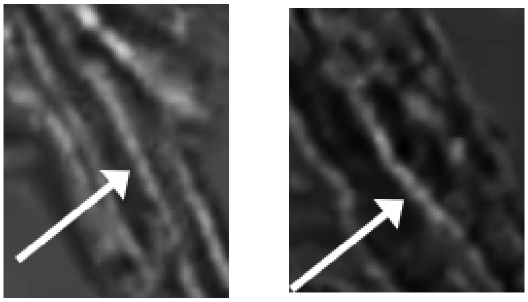

Fig. S2. M***yofibrils from failing ventricles are able to contract. Left panel:*** *myofibril from failing left ventricle in rigor.* ***Right panel****: The same myofibril 200 msec after adding contracting solution. The image has been contrast enhanced.*

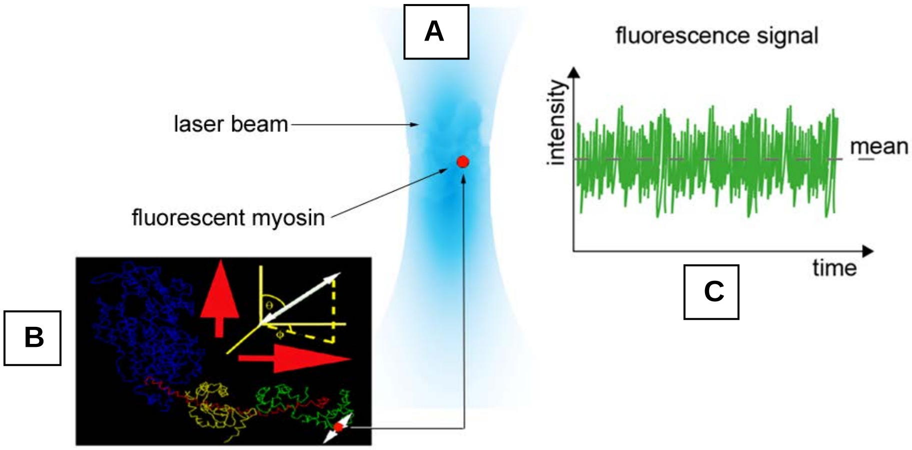

Fig. S3. **Top *panel: Origin of fluctuations.*** *The orientation of the combined transition dipole varies during contraction, because XB lever arm swings periodically as the lever arm assumes different configurations during actomyosin cycle. The position of myosin molecule (red dot in **A**) remains constant, but the angle of its absorption/emission dipole (shown as a white arrow in **B**) Thus the intensity fluctuations (green in **C**) are caused by rotational motion of the lever arm. From the fluctuations the kinetic constants are obtained by calculating autocorrelation function as explained below.*

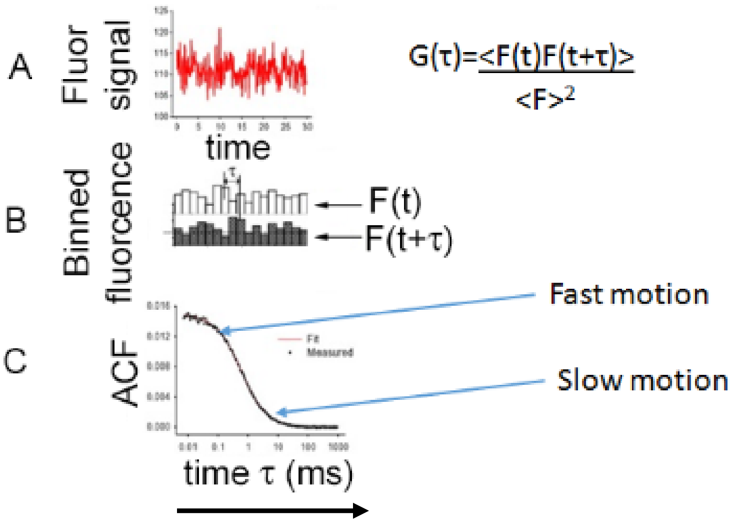

Bottom panel: Calculation of AutoCorrelation Function. *Fluorescence signal (A) is divided into 2,000 bins, each containing number representing polarization of fluorescence (normalized number of photons collected in ‖ channel minus photons collected in ⊥ channel). The numbers in the bins are multiplied by numbers in the "delayed" bin (shifted by increasing a delay time τ from 1msec to 1 sec (B)). The resulting ACF is shown in (C). The ACF value at a short delay time τ indicates a faster kinetic process, while at a longer delay time τ, indicates a slower process.*

Fig. S4 *Summary of kinetic data from contracting failing heart. Not Statistically Significant, Statistically Significant*

HF Contraction	k_{ADP}	k_{DISS}
HF 117DD LV	1.76±0.06	2.27±0.06
HF 117DD RV	1.78±0.09	2.32±0.16

HT Contraction	k_{ADP}	k_{DISS}
HF 43149 LV	1.71±0.05	2.27±0.07
HF 43149 RV	1.70±0.05	2.25±0.1

HF Contraction	k_{ADP}	k_{DISS}
HF 668C5 LV	1.74±0.08	2.30±0.10
HF 668C5 RV	1.75±0.06	2.74±0.08

HF Contraction	k_{ADP}	k_{DISS}
HF 98868 LV	1.69±0.07	2.23±0.10
HF 98868 RV	1.65±0.04	2.21±0.10

HF Contraction	k_{ADP}	k_{DISS}
HF 74B28 LV	1.75±0.09	2.31±0.12
HF 74B28 RV	1.72±0.05	2.45±0.13

Fig. S5. *Summary of FWHM data from contracting failing heart. For each ventricle the differences were not statistically significant.*

HF 117DD Contract FWHM	
LV	RV
0.375	0.406

HF 43149 Contract FWHM	
LV	RV
0.387	0.385

HF 668C5 Contract FWHM	
LV	RV
0.379	0.377

HF 98868 Contract FWHM	
LV	RV
0.388	0.386

HT 74B28 Contract FWHM	
LV	RV
0.406	0.384

Fig. S6.

We have data from two non-failing hearts. It is not included in the main text of communication because statistical analysis could not be carried out on 2 samples. The preliminary data obtained from 23 ventricular myofibrils obtained from a two non-failing human hearts is presented in **Table** below.

Contraction of ventricles from non-failing heart	k_{ADP} (s^{-1})	k_{DISS} (s^{-1})	FWHM
**LV**	3.9±2.7	0.4±0.15	0.360±0.041
**RV**	1.6±1.0	2.6±0.9	0.372±0.059

The average values of distribution of myosin lever arm orientations in thin filaments during contraction of 23 ventricular myofibrils from two non-failing human hearts.
